# Seroprevalence of Cytomegalovirus Among Pregnant Women at Kawempe National Referral Hospital, Uganda: A Cross-sectional Study

**DOI:** 10.1093/ofid/ofae604

**Published:** 2025-03-10

**Authors:** Richard V Katungye, Moses Musooko, Musa Sekikubo, Tobius Mutabazi, Mary Kyohere, Valerie Tusubira, Juliet Nsimire Sendagala, Joseph Peacock, Kirsty Le Doare, Annettee Nakimuli, PROGRESS Study Author Group, Abdelmajid Djennad, Agnes Nyamaizi, Agnes Ssali, Alexander Amone, Amusa Wamawobe, Annettee Nakimuli, Caitlin Farley, Carol Nanyunja, Christine Najuka, Cleophas Komugisha, Dan R Shelley, Edward A R Portal, Ellie Duckworth, Emilie Karafillakis, Geraldine O’Hara, Godfrey Matovu, Hannah G Davies, Janet Seeley, Joseph Peacock, Juliet Nsimire Sendagala, Katie Cowie, Kirsty Le Doare, Konstantinos Karampatsas, Lauren Hookham, Madeleine Cochet, Margaret Sewegaba, Mary Kyohere, Maxensia Owor, Melanie Etti, Merryn Voysey, Moses Musooko, Musa Sekikubo, Owen B Spiller, Patience Atuhaire, Paul T Heath, Philippa Musoke, Phiona Nalubega, Pooja Ravji, Richard Katungye, Ritah Namugumya, Rosalin Parks, Rose Azuba, Sam Kipyeko, Simon Beach, Stephen Bentley, Tim Old, Tobius Mutabazi, Valerie Tusubira, Vicki Chalker

**Affiliations:** Department of Obstetrics and Gynaecology, Makerere University College of Health Sciences, Kampala, Uganda; Directorate of Health Services, Special Forces Command, Uganda People's Defense Forces, Entebbe, Uganda; Department of Obstetrics and Gynaecology, Makerere University College of Health Sciences, Kampala, Uganda; Department of Obstetrics and Gynaecology, Makerere University College of Health Sciences, Kampala, Uganda; Directorate of Health Services, Special Forces Command, Uganda People's Defense Forces, Entebbe, Uganda; School of Medicine, Makerere University College of Health Sciences, Kampala, Uganda; Makerere University—Johns Hopkins University Research Collaboration, Kampala, Uganda; Institute for Infection and Immunity, St. George's, University of London, London, UK; Makerere University—Johns Hopkins University Research Collaboration, Kampala, Uganda; Medical Research Council/Uganda Virus Research Institute and London School of Hygiene & Tropical Medicine Uganda Research Unit, Entebbe, Uganda; Institute for Infection and Immunity, St. George's, University of London, London, UK; Makerere University—Johns Hopkins University Research Collaboration, Kampala, Uganda; Institute for Infection and Immunity, St. George's, University of London, London, UK; Medical Research Council/Uganda Virus Research Institute and London School of Hygiene & Tropical Medicine Uganda Research Unit, Entebbe, Uganda; Department of Obstetrics and Gynaecology, Makerere University College of Health Sciences, Kampala, Uganda

**Keywords:** CMV, infants, pregnant women, seropositivity, Uganda

## Abstract

**Background:**

Maternal primary cytomegalovirus (CMV) infection is associated with abortion and congenital anomalies. In Uganda, the burden of maternal CMV infection is not well studied. This study thus assessed the seroprevalence and factors associated with CMV infection among pregnant women at Kawempe National Referral Hospital in Kampala. This work forms a part of the PROGRESS study, an observational cohort study undertaken in Kampala, Uganda, between November 2018 and April 2021.

**Methods:**

We conducted a cross-sectional study between September 2020 and January 2021 among the 639 pregnant women admitted to the labor ward at a government hospital. Sociodemographic, medical, obstetric, and socioeconomic data were collected. Blood samples from study participants were drawn and analyzed for the presence of CMV immunoglobulin G (IgG) and IgM using enzyme-linked immunosorbent assay–based quantitative assays. Further analysis of all IgM-positive samples was conducted using CMV IgG avidity assays. All infants had a nasal polymerase chain reaction (PCR) on the first day of life to investigate CMV positivity. Logistic regression was performed to determine the factors associated with CMV infection.

**Results:**

Seroprevalence of CMV IgG among the 637 women was universal (100%), and that of CMV (IgM) was 5.8% (37/637). CMV (IgM) was associated with being low socioeconomic status (odds ratio, 3.44; 95% CI, 1.05–11.32; *P* = .04). Transmission risk was low, and no infant had a positive PCR for CMV at birth.

**Conclusions:**

Universally, by the time women in Kampala conceive, they will have been exposed to CMV. Women of lower socioeconomic status were more likely to have recent CMV infection than their more affluent counterparts, highlighting the need for screening guidelines in this setting.

Cytomegalovirus (CMV) is the leading cause of congenital infection worldwide [1]. Transplacental spread can occur at any gestational age and presents with a variety of abnormal pregnancy and birth outcomes, occurring in up to 30% of primary and 2% of recurrent CMV infections in pregnancy [2]. Congenital CMV (cCMV) can cause permanent sequelae in about 15% to 18% of births including neurocognitive sequelae, hearing loss, and infant mortality [3, 4].

CMV is ubiquitous in African countries, with seroprevalence in pregnancy ranging from 88% to 100% [5, 6] and a cCMV rate of ∼3% despite preexisting maternal immunity. It is strongly associated with maternal HIV infection [7]. The consequences for infants with cCMV have not been widely studied in Africa, but there is emerging consensus that cCMV may be a cause of significant morbidity and mortality.

Low socioeconomic status, poor sanitation, and poor housing conditions are among the factors associated with CMV infection [8, 9]. Uganda has a rapid annual population growth rate of about 3.3%, and the majority of people in urban centers are of low socioeconomic status, living in communities with poor housing and sanitation [10]. In these settings, the sero-prevalence of CMV among children aged <1 year is 83% [11], which contrasts with the rate observed in European countries, where CMV exposure occurs among older children.

Data on the magnitude of CMV among pregnant women in Uganda are limited despite the potentially significant cCMV burden among infants. Consequently, CMV screening services in antenatal care programs to detect mothers at high risk of congenital transmission have not been embraced. This study therefore determined the seroprevalence of CMV and associated factors among pregnant women and their infants at Kawempe National Referral Hospital.

This paper forms part of a supplement based on the PROGRESS study. The seroepidemiology of maternally derived antibodies against group B *Streptococcus* in Mulago/Kawempe Hospitals Uganda (PROGRESS) study aimed to describe the causes of infectious mortality and morbidity as well as the seroepidemiology of group B streptococcal infection—the major cause of neonatal sepsis worldwide—in Kampala, Uganda [12].

## METHODS

### Study Design

This was a cross-sectional study conducted between September 2020 and January 2021 among 637 pregnant women admitted to the labor ward at Kawempe National Referral Hospital. Participant recruitment sites for the studies that form part of this supplement are detailed in a flowchart available in the supplementary material of another paper published in this issue [13].

### Study Setting

Kawempe National Referral Hospital (KNRH) is a tertiary referral hospital in Kampala, located in central Uganda. Kampala city has a population of about 1 610 500; 827 797 are women [14]. KNRH offers free health care services and specializes in obstetrics, gynecology, pediatrics, and neonatal care services. On average, the facility conducts 70 labor ward admissions daily and 23 434 deliveries annually [15]. The highest percentage of patients admitted at the facility are low–socioeconomic status members of the community who reside in the city slums, which are characterized by poor sanitation and hygiene conditions. The labor ward is a very busy site that is run by a skilled team of health workers led by a specialist, with 3–5 residents, 1 medical officer, 2–3 intern doctors, and several cadres of midwives operating in shifts; it runs on a 24-hour basis.

### Study Population and Procedures

Pregnant women who were admitted to the labor ward at Kawempe National Referral Hospital between September 21, 2020, and January 15, 2021, and met the study eligibility criteria were systematically enrolled into the study. In order to randomly select participants, research assistants screened every 10th pregnant woman attending care for enrollment into the study. A participant was enrolled if she was aged 15–49 years, was pregnant and admitted to the labor ward, and gave full written informed consent to participate in the study. Research assistants collected data on sociodemographic characteristics (age, marital status, highest level of education, and wealth [determined by income and possession of household items]), medical characteristics (weight, height, gravidity, parity, mid-upper arm circumference [MUAC], body mass index [BMI; determined as a woman's weight in kilograms divided by the square of height in meters], history of hypertension, diabetes, tuberculosis, and HIV status), and behavioral characteristics (alcohol consumption and smoking status), which were recorded using Research Electronic Data Capture (REDCap) tools [16]. Venous blood samples from each participant were collected into Serum Separator Tube (SST) vacutainers. The sample was centrifuged on site and sent to the Medical Research Council–Uganda Virus Research Institute (MRC-UVRI) laboratory for CMV serology analysis. All infants had a nasal swab taken by a trained health care worker before discharge. Swabs were also sent to MRC-UVRI for real-time polymerase chain reaction (PCR).

### Laboratory Analysis

**Figure 1.**
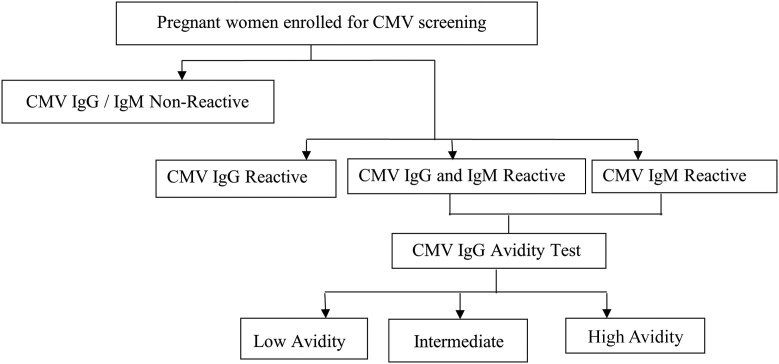
Algorithm for evaluation of cytomegalovirus infection in pregnancy. Abbreviations: CMV, cytomegalovirus; Ig, immunoglobulin.

Laboratory analyses were conducted at MRC-UVRI laboratory, which is accredited by the International Standards Organization (#ISO-15189-2012) and Good Clinical Laboratory Practices (GCLP) Accreditation Scheme-UK (#01908). CMV IgG and IgM were detected using the quantitative Elecsys CMV Immunoglobulin Assay. The assay detection range for IgG and IgM was 0.25–500 µ/mL, where the threshold for reactive titer was >1 µ/mL (specificity 96.6% and sensitivity 100% vs specificity 92.3% and sensitivity 93.9%, respectively). The CMV IgG low avidity index test was conducted on all IgM-positive samples using the Elecsys CMV IgG Avidity Assay. Low avidity was defined at <45%, indicating the highest risk of vertical transmission. Intermediate avidity ranged from 45% to 54.9%, and high avidity was defined at ≥55%, indicating the lowest risk of vertical transmission (assay sensitivity 94.8%, specificity 95.8%). All assays were run on a COBAS-601 analyzer, where daily quality control and calibration were performed. Infants born to women with a positive IgM were subjected to a CMV PCR test. The algorithm for evaluation of cytomegalovirus infection is presented in Figure 1.

### Data Management and Analysis

Univariate analyses were conducted to describe the sociodemographic and medical characteristics of the study population. Results were categorized as CMV negative or CMV positive. First, to determine the seroprevalence of CMV, which was defined as past exposure to CMV and indicated by the presence of CMV IgG antibodies in serum using a quantitative enzyme-linked immunosorbent assay (ELISA), we divided the number of pregnant women who tested CMV (IgG) positive by the total number of study participants. CMV IgG positive was defined as having CMV serum titer values ≥1.0 units/mL. Logistic regression analyses were conducted to determine factors associated with CMV among pregnant women. The primary outcome was a woman testing CMV IgM positive, defined as having a CMV serum titer cutoff index ≥1.0. All statistical tests were 2-sided with adjusted odds ratios that were presented with their 95% CIs and *P* values. At the multivariate level, statistical significance was considered for the factors whose *P* values were <.05. Analysis was undertaken using STATA, version 15 (Stata Corp LP, College Station, TX, USA).

## RESULTS

### Study Population

**Figure 2.**
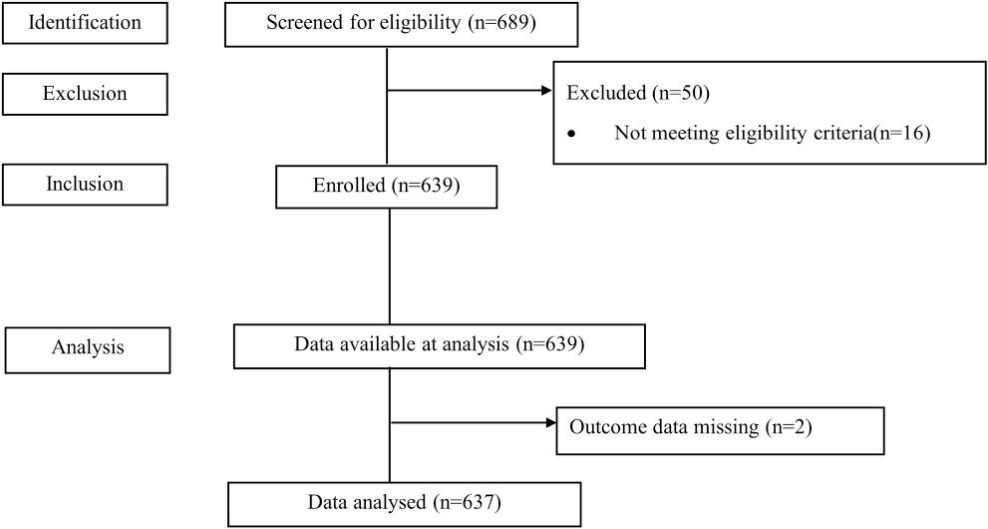
Study flowchart.

The study enrolled 639 pregnant participants, from whom 637 samples were analyzed. Two of the 639 samples were not included in the final analysis for having faint labels that could not be read. Details are illustrated in Figure 2. The median age (interquartile range) of the study participants was 24 (21–29) years, and 26.5% (169/637) of women had an MUAC <23 cm. The majority of participants (56.2%, n = 358) reported some secondary education. About 9% (55/637) of the study participants had a history of previous abortion. HIV prevalence among the study population was 4.6%. There was no congenital abnormality recorded. Details of the sociodemographic and medical characteristics of the study population are presented in Table 1.

**Table 1.** Sociodemographic and Medical Characteristics

Characteristic	Frequency, No. (%) n = 637	Outcome (CMV IgM)		95% CI
		Positive, No. (%)	Negative, No. (%)	
Age	n = 625			
≤24 y	335 (53.6)	22 (6.6)	313 (93.4)	0.43–0.77
25–34	257 (41.1)	13 (5.1)	244 (94.9)	0.19–0.51
≥35 y	33 (5.3)	1 (3.0)	32 (97.0)	0.001–0.15
Education level				
None	12 (1.9)	1 (8.3)	11 (91.7)	0.001–0.14
Primary	225 (35.3)	12 (5.3)	213 (94.7)	0.18–0.50
Secondary	358 (56.2)	19 (5.3)	339 (94.7)	0.34–0.68
Tertiary	42 (6.6)	5 (11.9)	37 (88.1)	0.05–0.29
Marital status				
Single	20 (3.2)	2 (10.0)	18 (90.0)	0.01–0.18
Married	427 (67.0)	25 (5.9)	402 (94.1)	0.50–0.82
Cohabiting	190 (29.8)	10 (5.26)	180 (94.74)	0.14–0.44
Wealth				
Very poor	389 (61.0)	21 (5.4)	368 (94.6)	0.39–0.73
Poor	122 (19.2)	12 (9.8)	110 (90.2)	0.18–0.50
Less poor	126 (19.8)	4 (3.2)	122 (96.8)	0.03–0.25
Body mass index				
Underweight	2 (0.3)	1 (50.0)	1 (50.0)	0.001–0.14
Normal	136 (21.3)	7 (5.15)	129 (94.85)	0.08–0.35
Overweight	324 (50.9)	22 (6.8)	302 (93.2)	0.42–0.75
Obese	175 (27.5)	7 (4.0)	168 (96.0)	0.08–0.35
MUAC				
≤23 cm (under nutrition status)	169 (26.5)	12 (7.1)	157 (92.9)	0.18–0.50
>23 cm (normal nutrition status)	468 (73.47)	25 (5.3)	443 (94.7)	0.50–0.82
Gravidity				
1	291	18 (6.2)	273 (93.8)	0.32–0.66
2–4	275 (43.2)	17 (6.2)	258 (93.8)	0.29–0.63
>4	71 (11.1)	2 (2.8)	69 (97.2)	0.01–0.18
Parity				
Low parity (≤4 pregnancies)	605 (95.0)	36 (6.0)	569 (94.0)	0.89–0.99
High parity (>4 pregnancies)	32 (5.0)	1 (3.1)	31 (96.9)	0.001–0.14
HIV status				
Positive	29 (4.6)	0 (0.0)	29 (100.0)	
Negative	608 (95.4)	37 (6.1)	571 (93.9)	
History of abortion				
No	582 (91.4)	37 (6.4)	545 (93.6)	
Yes	55 (8.6)	0 (0.0)	55 (100.0)	
History of diabetes				
No	614 (96.4)	36 (5.9)	578 (94.1)	0.89–0.99
Yes	23 (3.6)	1 (4.3)	22 (95.7)	0.001–0.14
History of hypertension				
No	613 (96.2)	36 (5.9)	577 (94.1)	0.89–0.99
Yes	24 (3.8)	1 (4.17)	23 (95.83)	0.001–0.14

Abbreviations: CMV, cytomegalovirus; Ig, immunoglobulin; MUAC, mid-upper arm circumference.

### Seroprevalence of CMV Infection and Associated Factors

The seroprevalence of CMV IgG was 100% (637/637), and the seroprevalence of CMV IgM was 5.8% (37/637). The IgG avidity index for all CMV IgM–positive samples was >45% (low risk of vertical transmission), and all infants born to women with positive IgM had a negative CMV PCR.

Pregnant women who were poor as compared with the less poor ones were about 3 times more likely to have CMV infection (adjusted odds ratio, 3.44; 95% CI, 1.05–11.32; *P* = .04). Details are summarized in Table 2.

**Table 2.** Factors Associated With CMV Infection Among Pregnant Women Admitted in Labor at Kawempe National Referral Hospital

Variable	Frequency n = 637	Positive IgM Outcome, n (%)	Negative IgM Outcome, n (%)	Bivariate Analysis, cOR (95% CI)	*P* Value	Multivariate Analysis, aOR (95% CI)	*P* Value
Age	n = 625						
≤24 y	335	22 (6.6)	313 (93.4)	Ref	…	Ref	
25–34 y	257	13 (5.1)	244 (94.9)	0.76 (0.37–1.54)	.44	0.69 (0.33–1.44)	.33
≥35 y	33	1 (3.0)	32 (97.0)	0.44 (0.06–3.42)	.44	0.06 (0.07–4.19)	.53
Education level							
None	12	1 (8.3)	11 (91.8)	0.67 (0.07–6.38)	.70	0.73 (0.07–7.31)	.79
Primary	225	12 (5.3)	213 (94.7)	0.42 (0.14–1.25)	.119	0.42 (0.12–1.21)	.14
Secondary	358	19 (5.3)	339 (94.7)	0.42 (0.15–1.18)	.098	0.33 (0.11–0.99)	.05
Tertiary	42	5 (11.9)	37 (88.1)	Ref	-	Ref	
Wealth status							
Very poor	389	21 (5.4)	368 (94.6)	1.74 (0.58–5.17)	.318	1.70 (0.54–5.34)	.36
Poor	122	12 (9.8)	110 (90.2)	3.33 (1.04–10.62)	.042	3.44 (1.05–11.32)	**.04**
Less poor	126	4 (3.2)	122 (96.8)	Ref		Ref	
BMI							
Normal	136	7 (5.1)	129 (94.9)	Ref		Ref	
Underweight	2	1 (50.0)	1 (50.0)	18.43 (1.04–326.42)	.05	24.50 (1.30–462.28)	**.03**
Overweight	324	22 (6.8)	302 (93.2)	1.34 (0.56–3.22)	.51	1.28 (0.52–3.14)	.59
Obese	175	7 (4.0)	168 (96.0)	0.77 (0.26–2.24)	.63	0.71 (0.24–2.14)	.55

Abbreviations: aOR, adjusted odds ratio; BMI, body mass index; cOR, crude odds ratio; Ig, immunoglobulin.

## DISCUSSION

The results from this study indicate universal CMV exposure in this population and low transmission risk. Several studies conducted among pregnant women in Africa have reported equally high levels of CMV exposure; 88.4% among pregnant women in Kenya [5], 96% among Egyptian women [6], 88.6% in Ethiopia [17], 99.6% in Zimbabwe [18], 100% in Namibia [19], and 73.9% among pregnant women attending antenatal clinics in Tanzania [20]. This low risk of vertical transmission was supported by the negative PCR results in infants, indicating that cCMV may not be vertically acquired in our population and early postnatal transmission may account for the high seropositivity rate by the age of 1 year in Uganda [11].

In this study, pregnant women in the lower wealth percentile were 3 times more likely to have recent CMV infection compared with the highest percentile. These findings are consistent with previous studies conducted in the United States and Canada that found a higher burden of CMV among people of low socioeconomic status compared with those with a higher status [9, 21–23]. The probable reason for the higher prevalence of CMV infection among the poor could be due to poor hygiene standards among the urban poor population. Increased risk of CMV exposure has previously been associated with poor standards of living [24, 25]. It is notable that part of the catchment area of our study has densely populated slums characterized by poor housing and sanitation facilities, which could explain the extremely high risk of CMV exposure among the study population.

### Limitations

This study acknowledges several limitations. A single CMV ELISA titer may not offer a sufficiently conclusive result; hence serial titers may be required for confirmation, although the study was not designed to follow women longitudinally. No formal neurodevelopmental assessment of infants occurred, so we cannot completely rule out neurodevelopmental sequelae.

## CONCLUSIONS

Despite universal CMV IgG seroprevalence, a moderate proportion of 5.8% had CMV infection in pregnancy, and this was common among the poor population of women. Our findings demonstrate a need for better screening policies for women and children to investigate the true burden of cCMV in Uganda.

## Notes

***Acknowledgments.*** We would like to acknowledge St George's, University of London, in collaboration with MUJHU and UPDF Chieftaincy of Medical Services, for sponsorship of this research. We would also like to note our appreciation for the study staff Cleophas Komugisha, Ritah Kukundakwe, Prossy Nagujja, Florence Nambazira, and Godfrey Matovu. Our sincere gratitude goes to all study participants for their time and participation.

The complete PROGRESS Study Author Group is comprised of Abdelmajid Djennad, Agnes Nyamaizi, Agnes Ssali, Alexander Amone, Amusa Wamawobe, Annettee Nakimuli, Caitlin Farley, Carol Nanyunja, Christine Najuka, Cleophas Komugisha, Dan R. Shelley, Edward A. R. Portal, Ellie Duckworth, Emilie Karafillakis, Geraldine O’Hara, Godfrey Matovu, Hannah G. Davies, Janet Seeley, Joseph Peacock, Juliet Nsimire Sendagala, Katie Cowie, Kirsty Le Doare, Konstantinos Karampatsas, Lauren Hookham, Madeleine Cochet, Margaret Sewegaba, Mary Kyohere, Maxensia Owor, Melanie Etti, Merryn Voysey, Moses Musooko, Musa Sekikubo, Owen B. Spiller, Patience Atuhaire, Paul T. Heath, Philippa Musoke, Phiona Nalubega, Pooja Ravji, Richard Katungye, Ritah Namugumya, Rosalin Parks, Rose Azuba, Sam Kipyeko, Simon Beach, Stephen Bentley, Tim Old, Tobius Mutabazi, Valerie Tusubira, and Vicki Chalker.

***Author contributions*.** R.V.K., K.L.D., and A.N. conceived the study. A.N., K.L.D., R.V.K., M.S., and M.M. designed the study. T.M., V.T., J.N.S., and M.K. were responsible for software, data collection, data curation, and formal analysis. R.V.K. and T.M. drafted the original manuscript. A.N., K.L.D., M.K., M.S., M.M., J.P., and V.T. reviewed and edited the manuscript. A.N. and K.L.D. supervised the project. All authors reviewed and approved the final manuscript.

***Ethics approval and consent to participate*.** Ethical approval (#REC REF 2020-114) was obtained from the School of Medicine Research and Ethics Committee (SOMREC) of Makerere University and Uganda National Council of Science and Technology (UNCST), as well as St George's, University of London (SGUL REC ref 2020.0024). At the health facility, informed consent was sought from all study participants, and confidentiality of research data was ensured by all the research assistants.

***Availability of data and materials*.** The data sets used and/or analyzed in this study are available from the corresponding author on reasonable request.

***Data availability*.** The full data set for the PROGRESS study is available in Le Doare [26].

***Financial support***. This work was supported by the Bill and Melinda Gates Foundation (OPP1189053) and sponsored by St George's, University of London (SGUL), R&D Protocol Code Number 17.0018. The funder had no role in the study design, data collection and analysis, decision to publish, or preparation of the manuscript. The funders had no access to the data set of this study.

***Supplement sponsorship***. This article appears as part of the supplement “Neonatal and Young Infant Infection in Uganda: the PROGRESS Study,” sponsored by St George's, University of London.
